# Diazotrophic Community Structure and Environmental Correlates in BSCs Under Sand-Fixing Plantations in Alpine Sandy Land

**DOI:** 10.3390/microorganisms14091914

**Published:** 2026-08-29

**Authors:** Xionglian Jin, Huichun Xie, Xiaoping Kong, Jiawei Yan, Yonggui Ma, Zhe Chen, Feng Qiao

**Affiliations:** 1 College of Geographical Sciences, Qinghai Normal University, Xining 810008, China; 18997124460@163.com; 2 School of Life Sciences, Qinghai Normal University, Xining 810008, China; 3 Key Laboratory of Medicinal Animal and Plant Resources of the Qinghai-Tibetan Plateau, Xining 810008, China; 4 Research Team on Formation Mechanism and Utilization of Germplasm Resources on the Qinghai-Tibetan Plateau, Xining 810008, China; 5 Qilian Mountain Southern Slope Forest Ecosystem Research Station, Huzhu 810500, China; 6 Xining Botanical Garden, Xining 810008, China

**Keywords:** alpine sandy land, biological soil crusts (BSCs), *nifH* diazotrophic community, soil factors, responding to change

## Abstract

Biological soil crusts (BSCs) are critical components of alpine sandy land ecosystems, yet how their diazotrophic communities respond to plantation type and successional stage remains largely unknown. This study examined diazotrophic community differentiation in BSCs under four sand-fixing plantations (*Salix psammophila* SL, *Caragana korshinskii* NT, *Salix cheilophila* WL, *Populus simonii* XYY) across three successional stages in an alpine sandy land. Soil properties, microbial biomass, and enzyme activities were measured, along with *nifH* gene sequencing (Illumina), to characterize community composition and its relationships with environmental factors along BSCs succession. The results indicate that Skermanella, Mastigocladus, and Nostoc were the dominant diazotrophic groups in the study area. The Nostoc was the dominant genus across all plantation types, with the highest average relative abundance (37.92%) recorded in moss crusts under the XYY plantations. Diazotrophic α-diversity increased with BSCs succession, and β-diversity analysis (PCoA with ANOSIM) demonstrated significant community differentiation among plantation types (variance explanation = 50.91%, *R* = 0.59458, *p* = 0.001). Spearman’s and redundancy analysis (RDA) showed that sand-fixing plantations drive the differentiation of diazotrophic communities by regulating soil physicochemical properties and microbial metabolism. Furthermore, the diazotrophic communities in moss crusts exhibited stronger environmental responsiveness than those in algae crusts. In the COG functional categories, J (translation and ribosomal biogenesis, 7.92%), E (amino acid metabolism, 10.14%), and C (energy production, 7.11%) were identified as the core foundational functions of BSCs in the study area. The relative abundances of these functional categories showed convergence. In summary, different plantations were associated with distinct diazotrophic community compositions, and these associations were largely mediated by soil environmental factors. Concurrently, BSC succession covaried with enhanced differentiation in community structure and functional traits. These patterns are consistent with a stepwise response cascade linking vegetation and soil properties to diazotrophic communities and nitrogen metabolism functions. This study provides a theoretical basis for enhancing the stability and long-term restoration of artificial ecosystems by regulating diazotrophic communities in BSCs in alpine sandy land.

## 1. Introduction

Biological soil crusts (BSCs) are organic composite systems formed through the synergistic interaction of autotrophic and heterotrophic organisms—such as soil microorganisms, algae, lichens, and mosses—with soil particles. They are widely distributed in desert ecosystems in arid and semiarid regions [1,2]. As an integral part of the sandy ecosystem, BSCs play a vital role in soil and water conservation, windbreak and sand fixation, as well as biological carbon and nitrogen sequestration [3,4,5]. In particular, algae crusts in the early stages of crust formation play a significant role in soil nitrogen input in desert ecosystems [6]. According to statistics, BSCs sequester approximately 7% of the carbon and more than 40% of the biological nitrogen in the world’s terrestrial ecosystems, contributing to 7% of primary productivity [7]. Biological nitrogen fixation is the process by which nitrogenase reduces N_2_ to ammonia, serving as the primary source of bioavailable nitrogen in nature [4]. The *nifH* gene encodes the iron protein subunit of nitrogenase and is highly conserved across diazotrophs. However, because diazotrophs are phylogenetically diverse and distributed across multiple phyla, they cannot be effectively targeted as a functional group using universal 16S rRNA gene primers [8]. The *nifH* gene contains both conserved and variable regions, and phylogenies inferred from *nifH* sequences are highly consistent with those derived from 16S rRNA genes [9,10]. For these reasons, *nifH* has been widely adopted as a molecular marker for investigating diazotrophic diversity.

In recent years, significant progress has been made in research on diazotrophic microbial communities in BSCs. Current research on deserts and sandy lands has shown that, at the phylum level, Cyanobacteria and Proteobacteria are the dominant groups, while at the genus level, Azospirillum and Nostoc predominate [11,12]. With regard to succession patterns, studies have also shown that the diversity and taxonomic richness of diazotrophic bacteria increase significantly as BSCs progress through their developmental stages [4]. Furthermore, the positive development of BSCs can significantly increase soil nitrogen accumulation, promoting ecosystem succession in sandy ecosystems [11]. Simultaneously, changes in soil physicochemical properties (pH, organic matter, total nitrogen, and moisture) driven by BSC succession restructure the microhabitat, leading to environmental filtering and species reshuffling that collectively shape the diazotrophic microbial community. Among these, total nitrogen and pH are key environmental factors that regulate the composition of diazotrophic microbial communities [11,13]. In addition, the establishment of artificial vegetation can significantly alter the microenvironment of the topsoil, increasing the coverage of BSCs and the abundance of diazotrophic genes. This, in turn, improves nitrogen cycling and nutrient accumulation in sandy soils [14]. As a critical component of the ecological security barrier, the alpine sandy land is inherently fragile, making artificial vegetation restoration essential for its ecological rehabilitation [15,16]. Current studies on BSCs under the sand-fixing plantations in the alpine sandy land of our study area have largely focused on the effects of BSC development on soil physicochemical properties and bacterial community structure [17,18]. However, the alpine sandy land differs from temperate deserts in its low mean temperature, freeze–thaw cycles, high UV radiation, and a short growing season [19,20,21]. These factors are expected to exert distinct environmental filters on diazotrophic communities. Despite these mechanistic expectations, no studies have been reported on diazotrophic microorganisms in BSCs under sand-fixing plantations in alpine sandy land. Furthermore, existing studies have elucidated the composition and environmental drivers of diazotrophic communities in sandy BSCs, but most have focused on temperate deserts and low-altitude wind-blown sandy areas [2,4,13]. Systematic studies targeting the extreme habitats of alpine sandy lands remain scarce. Under high-altitude cold environmental stress, the unique structure of diazotrophic communities in BSCs growing beneath various types of sand-fixing plantations, the distribution of dominant taxa, and the patterns of their differential responses remain unclear.

Despite these advances, the response of diazotrophic communities to plantation type and BSC succession in the alpine sandy land remains poorly understood. It is unclear whether these factors shape community structure, environmental drivers, and functional potential. To address this gap, this study tested whether diazotrophic diversity and composition shift with BSC succession, environmental drivers differ among plantation types, and nitrogen metabolism potential increases with succession. This study focused on BSCs growing under different sand-fixing plantations in alpine sandy land over a 30-year restoration period. By using high-throughput sequencing technology targeting the *nifH* functional gene, the species composition, diversity, and functional categories of diazotrophic communities in BSCs across different plantation types were systematically analyzed. The aim was to elucidate the relationship between soil environmental factors and diazotrophic communities across BSC successional stages. This study aimed to provide a microbiological basis for the efficient restoration of alpine sandy land ecosystems and the enhancement of soil nitrogen pools.

## 2. Materials and Methods

### 2.1. Description of the Study Area

**Figure 1:**
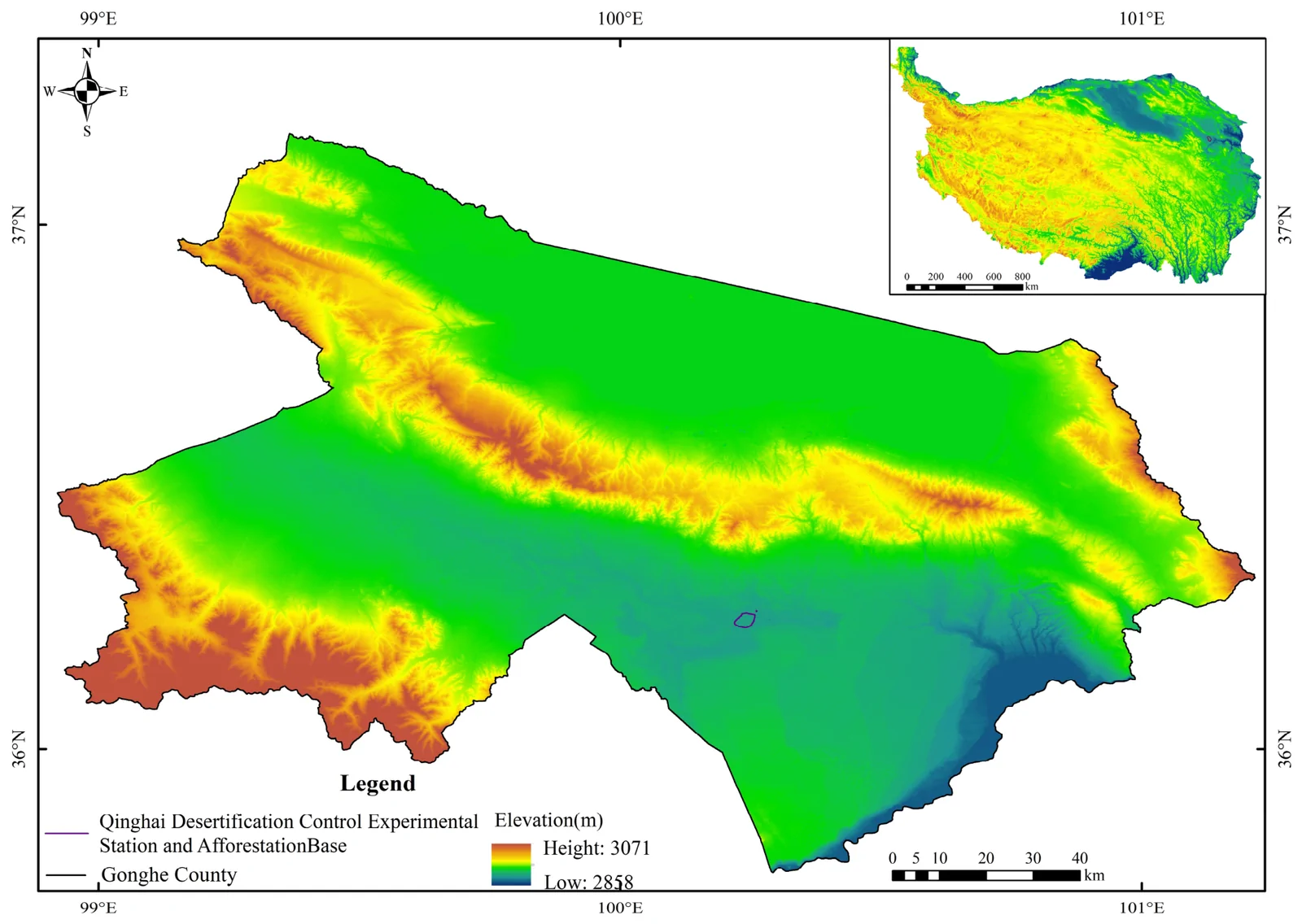
Location of the study area and sampling sites in the Gonghe Basin, Qinghai Province, China. Note: the purple circle indicates the Qinghai Desertification Control Experimental Station, within which the four sand-fixing plantation sites are located. Exact coordinates of each site are provided in Table 1.

The study area, the Gonghe Basin, is located on the northeastern edge of the Qinghai–Tibet Plateau and is part of Gonghe County, Hainan Tibetan Autonomous Prefecture, Qinghai Province (Figure 1). The basin is flanked by the Qilian, Kunlun, and Qinling orogenic belts, adjacent to the Qaidam Basin to the west and connected to the Guide Basin to the east [22]. The geographic coordinates are: 99°45′–100°30′ E, 36°03′–36°40′ N. The terrain is generally flat, comprising a mosaic of plains, ravines, and hills, with relatively gentle topographic relief. The elevation is relatively low, with elevations ranging from below 3000 m to a maximum of 4000 m. The region is sensitive to climate and environmental changes, with an average annual temperature of 2.0–3.3 °C, annual sunshine hours of 2600–2800 h, and average annual precipitation of 311.1–423.1 mm (concentrated from June to September). It falls within a cold, semi-arid climate zone at high altitude. In the early stages, the basin’s soils were classified into alpine and steppe soil series, encompassing 6 soil orders and 14 suborders [23]. Representative plant species include *Stipa krylovii*, *Stipa purpurea*, *Artemisia desertorum*, and *Orinus thoro* [24]. The location map (Figure 1) was created using ArcGIS software (v 10.8). The base map was generated from a 90-m-resolution DEM [25]. Administrative and land-use data for Gonghe County were obtained from the Resource and Environmental Science Data Center of the Chinese Academy of Sciences (https://www.resdc.cn), while the coordinates of the sampling plots were provided by the Qinghai Desertification Control Experimental Station.

### 2.2. Collection of BSC Samples

Field surveys and sample collection were conducted during the optimal growth period for BSCs (early to mid-October 2025) at the Qinghai Province Desertification Control Station in Shazhuyu Township, Gonghe Basin. Four representative sand-fixing plantation types, all established in 1990, were selected as sampling sites: *Salix psammophila* (SL), *Caragana korshinskii* (NT), *Salix cheilophila* (WL), and *Populus simonii* (XYY). The basic information on the sample plot is as follows (Table 1). Within each plantation type, three 60 m × 60 m large plots were randomly established as independent replicates, with a minimum spacing of >50 m between plots to ensure spatial independence. Within each large plot, three surface cover types were identified: bare sand (control), algae crusts, and moss crusts. For each cover type, six 6 m × 6 m subplots were randomly selected to estimate crust cover and thickness. Surface soil samples (0–2 cm depth) were then collected from each plot using a random multi-point sampling method. This design yielded a total of 36 independent samples (4 plantation types × 3 plots × 3 crust types), with each plot considered as the primary experimental unit for plot-level comparisons. For each composite sample, three aliquots were transferred into sterile 5-mL centrifuge tubes, preserved on dry ice in the field, and transported to the laboratory for subsequent analyses of physicochemical properties and diazotrophic communities. To avoid cross-contamination, disposable latex gloves were worn and changed between samples, and sampling tools were sterilized with 75% ethanol before each use. Each soil sample was placed into a sterile resealable bag, sealed, and clearly labeled with a unique sample ID. GPS coordinates, elevation, vegetation composition, and cover were recorded for each plot, and representative field photographs were taken to document BSCs at different successional stages.

**Figure 2:**
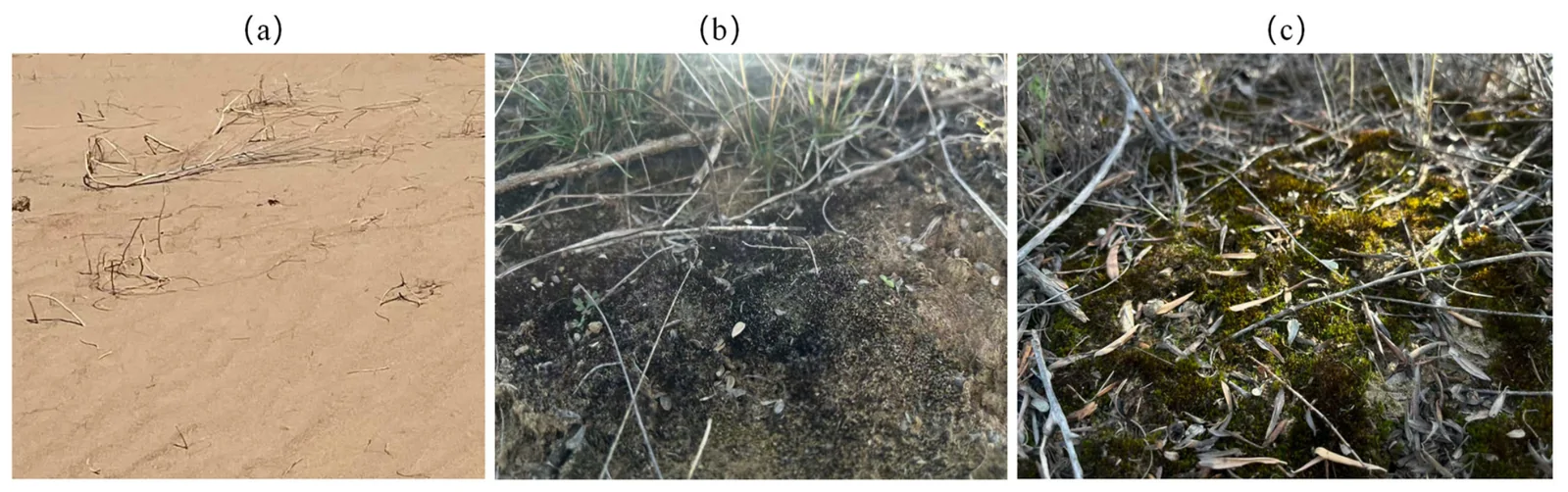
Field photographs of three types of land cover. Note: (**a**) bare sand; (**b**) algae crust; (**c**) moss crust.

Three surface cover types were selected: bare sand, algae crust, and moss crust. Bare sand served as the control, reflecting the original shifting sand substrate. Algae and moss crusts were designated as two BSC types at contrasting successional phases. Field photographs of the three types of land cover are shown below (Figure 2). Three surface types were defined based on field morphology according to previous classification criteria [26,27]: (1) Bare sand: no distinct crust, loose shifting sand; (2) algae crust: thin (1–3 mm), dark crust with cyanobacteria and green algae, filamentous structures visible; (3) moss crust: thick (5–15 mm) and dense, dominated by bryophytes (*Bryum* and *Didymodon*), green moss plants visible to the naked eye. The basic information on the samples is as follows (Table 2).

### 2.3. Determination of Physicochemical Factors in BSCs

To characterize soil nitrogen availability, ammonium nitrogen (NH_4_^+^-N) and nitrate nitrogen (NO_3_^−^-N) were determined. Extraction was performed using 1.00 mol/L KCl (Sinopharm, Shanghai, China). After shaking and filtering, the sample was analyzed using an AA3 continuous-flow analyzer (SEAL Analytical GmbH, Norderstedt, Germany). The samples were color-developed using salicylate-DCI (Sinopharm, Shanghai, China) and quantitatively analyzed by spectrophotometry at a wavelength of 660 nm. The results were used to calculate ammonium nitrogen (NH_4_^+^-N) after correction for the water-to-soil ratio and moisture content [28]; extraction was performed using 1.00 mol/L KCl (Shanghai, China), and the mixture was shaken and filtered. The resulting filtrate was then analyzed using an AA3 flow analyzer (Norderstedt, Germany). After reduction with alkaline hydrazine sulfate and color development with sulfanilamide-NEDD (Sinopharm, Shanghai, China), the sample was quantitatively analyzed by spectrophotometry at 550 nm. The results were used to calculate nitrate nitrogen (NO_3_^−^-N) after correction for the water-to-soil ratio and moisture content [29].

To assess microbial abundance, soil microbial biomass carbon (MBC), nitrogen (MBN), and phosphorus (MBP) were measured using the chloroform fumigation-extraction method. Fresh soil samples were divided equally into two groups: one group was subjected to vacuum fumigation with chloroform (Sinopharm, Shanghai, China) for 24 h, while the other group was not fumigated. Both samples were extracted with 0.50 mol/L K_2_SO_4_ (Sinopharm, Shanghai, China), shaken for 30 min, and then filtered. The ammonium nitrogen content in the extract was determined using an AA3 continuous-flow analyzer (Norderstedt, Germany). Divide the difference between the fumigated and unfumigated samples by 0.25 to obtain microbial biomass nitrogen (MBN) [30]; fresh soil samples were divided equally into two groups: one group was fumigated with chloroform (Shanghai, China) for 24 h, while the other group was not fumigated. Both samples were extracted with 0.50 mol/L K_2_SO_4_ (Shanghai, China), shaken for 30 min, and then filtered. The organic carbon content of the extract was determined using a TOC-VCPH organic carbon analyzer (Shimadzu, Japan). Divide the difference between the fumigated and unfumigated samples by 0.45 to obtain the microbial biomass carbon (MBC) [31,32]; fresh soil samples were divided equally into two groups: one group was subjected to 24 h of vacuum chloroform fumigation, while the other group was not fumigated. Both samples were extracted with 0.50 mol/L NaHCO_3_ (Sinopharm, Shanghai, China), shaken for 30 min, and then filtered. After the extract was color-developed using a molybdenum-antimony reagent, it was quantitatively analyzed by spectrophotometry at 882 nm. Divide the difference in phosphorus levels between the fumigated and unfumigated samples by 0.4 to obtain the microbial phosphorus (MBP) [33,34].

The soil physicochemical properties (including pH, soil water content (SWC), soil organic matter (SOM), alkaline-hydrolyzable nitrogen (AN), total nitrogen (TN), total carbon (TC), available phosphorus (AP), and available potassium (AK)) and enzyme activities (catalase (CAT), urease (URE), alkaline phosphatase (ALP), and sucrase (SUC)) analyzed in this study were obtained from the same samples as those reported in our previous work [35], where the detailed analytical procedures are described. While Reference [35] focused on the total bacterial community via 16S rRNA gene sequencing, the present study specifically targets the diazotrophic community using the *nifH* functional gene as a molecular marker—a fundamentally different scientific perspective and research question. Accordingly, the methodological details for these assays are not repeated here, and these data are subsequently used in correlation analyses with soil environmental variables.

### 2.4. DNA Extraction and Sequencing of Diazotrophic Community in BSCs

Weigh out 0.50 g of a soil sample stored at −80 °C and extract total DNA using the DNeasy PowerSoil Pro Kit (QIAGEN, Hilden, Germany). The integrity of the extracted total soil DNA was assessed by 1% agarose gel electrophoresis (Sinopharm, Shanghai, China) to determine whether it met the requirements for subsequent PCR amplification. DNA concentration and purity were determined using a NanoDrop 2000 UV-Vis spectrophotometer (Thermo Fisher Scientific, Wilmington, DE, USA). The A260/A280 and A260/A230 ratios were recorded to determine whether contaminants such as proteins or humic acids were present. Using the extracted total soil DNA as a template, PCR amplification was performed using universal primers for the nitrogenase *nifH* gene. PCR amplification was performed using the primer pair *nifH*-F (5′-AAAGGYGGWATCGGYAARTCCACCAC-3′) and *nifH*-R (5′-TTGTTSGCSGCRTACATSGCCATCAT-3′) [36]. The PCR reaction mixture is as follows: DNA template, forward and reverse primers, dNTPs, DNA polymerase, and the corresponding buffer. The PCR reaction conditions are as follows: 3-min pre-denaturation at 95 °C; 30-s denaturation at 94 °C, 30-s annealing at 55 °C, and 45-s extension at 72 °C, repeated for a total of 27 cycles; followed by a 10-min final extension at 72 °C. After verifying the target band in the PCR amplification product via 1.5% agarose (Shanghai, China) gel electrophoresis, the PCR product was purified using a gel recovery kit (Thermo Fisher Scientific, USA). The purpose is to remove impurities such as primer dimers, dNTPs, and enzymes. The purified amplicons were then used for library construction, and quantitative quality control of the library was performed using a Qubit fluorometer and real-time quantitative PCR (Thermo Fisher Scientific, USA). The prepared library was sequenced using paired-end sequencing on the Illumina MiSeq high-throughput sequencing platform. High-throughput sequencing analysis was performed by Shanghai Majorbio Bio-Pharm Technology Co., Ltd. (Shanghai, China).

### 2.5. Bioinformatic Analysis

Raw paired-end reads were quality-filtered and trimmed using fastp (v 0.20.0) on the Majorbio Cloud Platform. Reads with an average quality score below Q20 (i.e., <1% error probability) or a length shorter than 200 bp were discarded. Adapter sequences were removed, and overlapping paired-end reads were joined using FLASH (v 1.2.11) with a minimum overlap of 10 bp and a maximum mismatch rate of 10%. The joined sequences were clustered into operational taxonomic units (OTUs) at 97% sequence similarity using UPARSE (v 7.1). Chimeric sequences were identified and removed using UCHIME (v 4.2). Representative sequences for each OTU were selected, and taxonomic assignment was performed using the Majorbio *nifH* reference database (based on NCBI sequences). To infer the functional potential of the diazotrophic communities, COG (Clusters of Orthologous Groups of proteins) annotation was performed on the OTU representative sequences using the Majorbio Cloud Platform against the NCBI COG database (https://www.ncbi.nlm.nih.gov/research/cog-project/ accessed on 21 July 2026). This approach directly assigns query sequences to known functional categories based on sequence similarity. COG categories related to nitrogen metabolism were extracted for downstream analysis.

### 2.6. Statistical Analysis

Differences in inorganic nitrogen content (NH_4_^+^-N and NO_3_^−^-N), microbial biomass (MBC, MBN, and MBP), and α-diversity indices among plantation types and crust types were tested using one-way ANOVA followed by LSD post-hoc tests (SPSS 27.0, IBM, New York, NY, USA), with *p* < 0.05 considered statistically significant. Data organization and graphical presentation of these parameters were performed using WPS Spreadsheets (v 12.1.0.20784). Using the OTU clustering results, the community composition of diazotrophic bacteria was analyzed at the phylum and genus levels [37]. Principal coordinate analysis (PCoA) based on Bray–Curtis distances was used to visualize community separation patterns, and ANOSIM (analysis of similarities) was employed to test the significance of between-group differences [38]. The Kruskal–Wallis rank-sum test and one-way ANOVA were used to test for significant differences between groups at the genus level [39]. Redundancy analysis (RDA) at the OTU level and Spearman’s correlation analysis of the top 10 most abundant genera were performed to assess the associations between the diazotrophic community structure and environmental factors [40,41]. COG-based functional annotation of the diazotrophic communities was performed based on *nifH* amplicon sequencing data. COG annotation was conducted on OTU representative sequences to provide a coarse-grained functional categorization, with a focus on nitrogen metabolism-related categories [42]. The relative abundances of COG functional categories were calculated for each sample, and the resulting functional profiles were visualized to compare differences across plantation types and BSC successional stages. All figures presenting the analysis of the *nifH* gene-based diazotrophic communities were created using R (v 3.3.1).

## 3. Results

### 3.1. Inorganic Nitrogen and Microbial Biomass in BSCs

**Figure 3:**
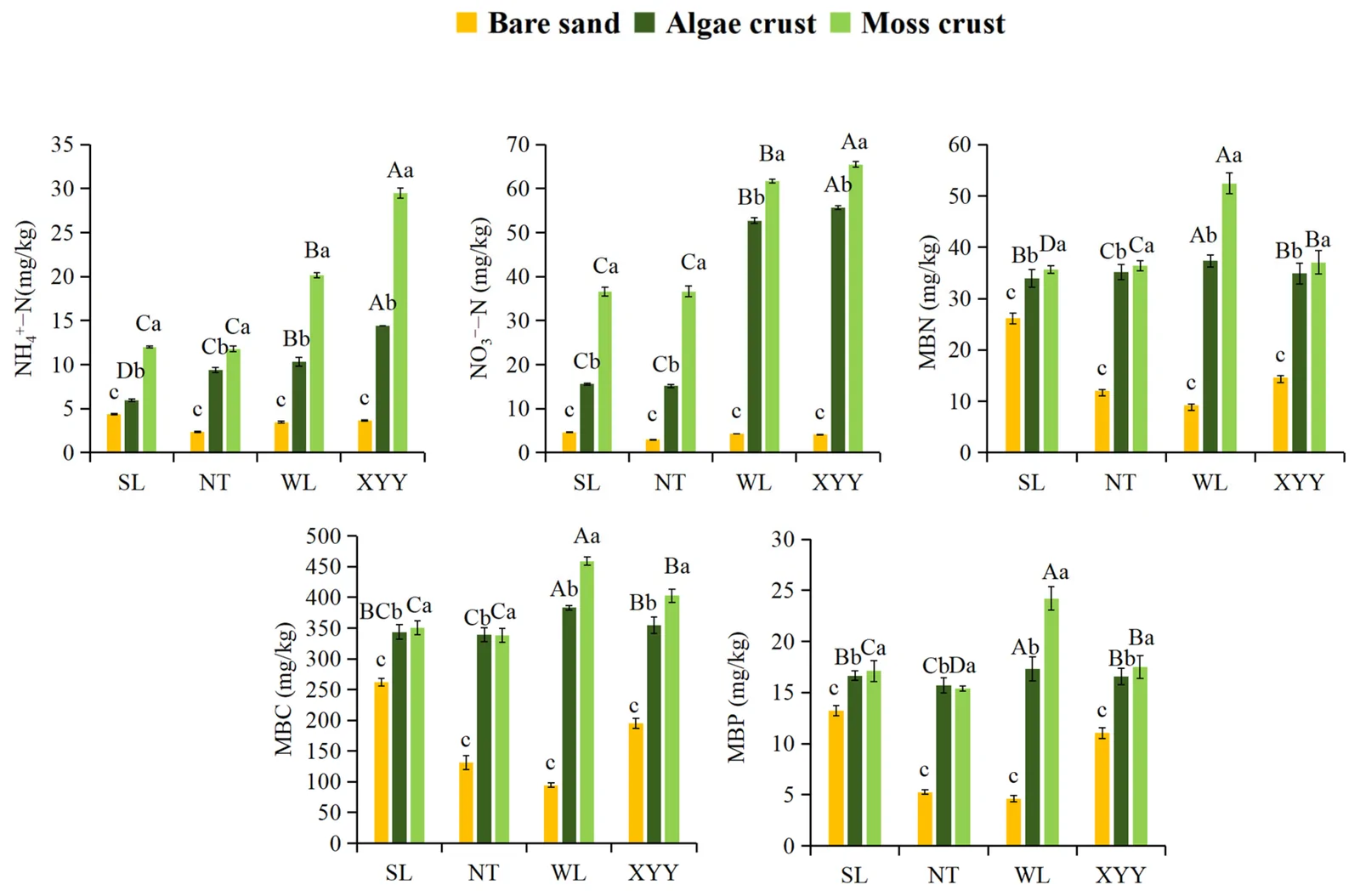
Inorganic nitrogen and microbial biomass in BSCs. Note: different uppercase letters indicate significant differences (*p* < 0.05) in the parameters of the same bark type across different plots, whereas different lowercase letters indicate significant differences (*p* < 0.05) in the parameters of different bark types within the same plot; SL: *Salix psammophila*; NT: *Caragana korshinskii*; WL: *Salix cheilophila*; XYY: *Populus simonii;* NH_4_^+^-N: ammonium nitrogen; NO_3_^−^-N: nitrate nitrogen; MBN: microbial biomass nitrogen; MBC: microbial biomass carbon; MBP: microbial biomass phosphorus.

Inorganic nitrogen content and microbial biomass in BSCs varied significantly among the four plantation types (Figure 3). Both the inorganic nitrogen content and microbial biomass in the BSCs were significantly higher than those in bare sand (ANOVA: F_2,33_ = 32.28–66.24, all *p* < 0.001, partial η^2^ = 0.66–0.80). As BSCs developed, both inorganic nitrogen content and microbial biomass showed an increasing trend, with the following order: moss crust > algae crust > bare sand. A further comparison among plantations of inorganic nitrogen and microbial biomass in algae and moss crusts is shown below. Among the four plantations, WL exhibited the highest MBC, MBN, and MBP. In the algae crust, these values were 383.06, 37.33, and 17.33 mg/kg, respectively; in the moss crust, they were 458.66, 42.42, and 24.20 mg/kg. In contrast, XYY showed the highest inorganic nitrogen. The NH_4_^+^-N and NO_3_^−^-N concentrations in the algae crust were 14.42 and 55.70 mg/kg, while in the moss crust they were 29.46 and 65.48 mg/kg. The development of BSCs significantly increase soil inorganic nitrogen content and microbial biomass. By enhancing nitrogen availability and microbial activity, it effectively improves soil fertility and promotes the restoration of the ecological functions of the topsoil.

### 3.2. Composition of the Diazotrophic Community in BSCs

**Figure 4:**
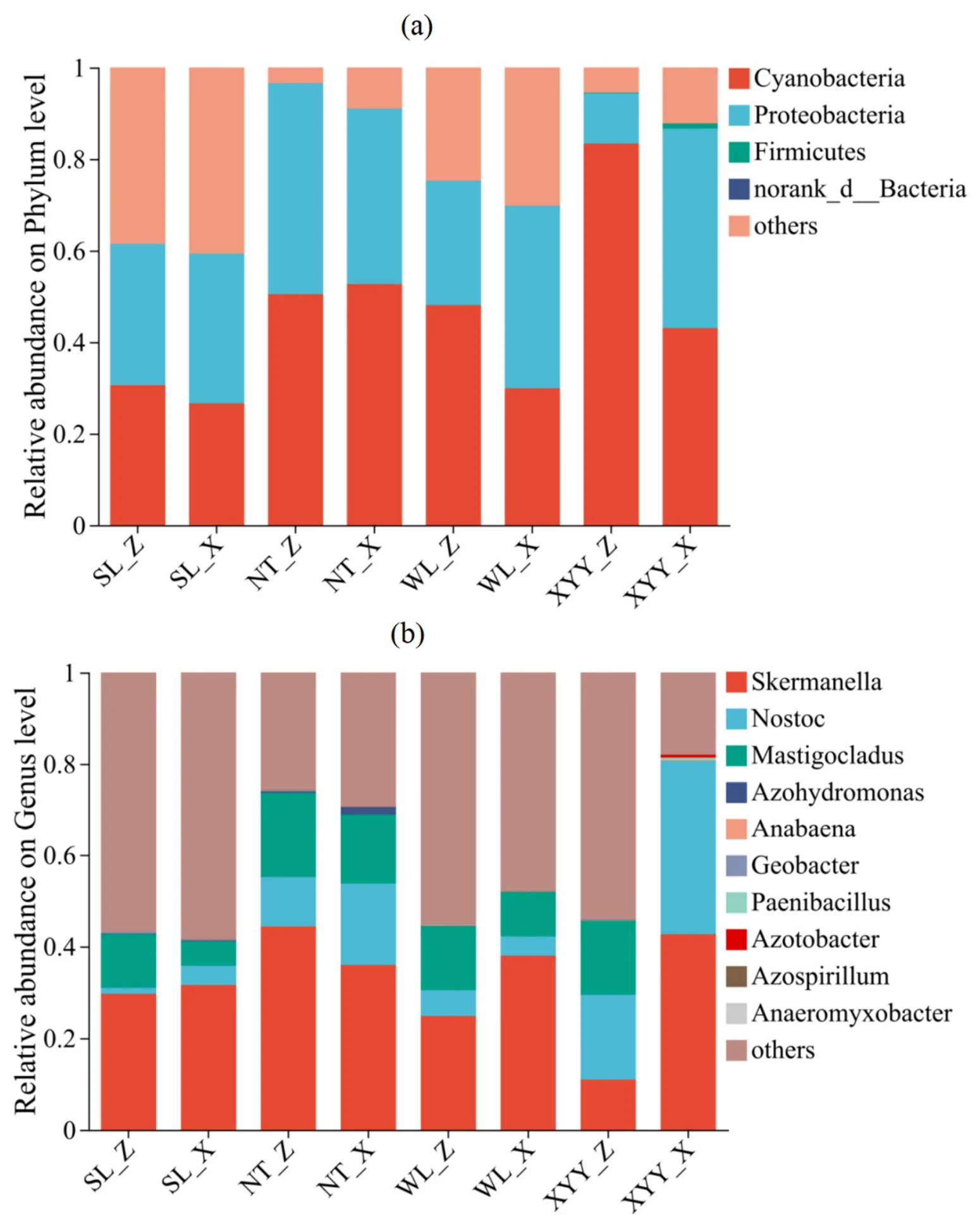
Composition of the diazotrophic community in BSCs. Note: show only the top 10 diazotrophic dominant groups ranked by relative abundance; (**a**) phylum-level composition of the *nifH* diazotrophic community in BSCs; (**b**) genus-level composition of the *nifH* diazotrophic community in BSCs; SL: *Salix psammophila*; NT: *Caragana korshinskii*; WL: *Salix cheilophila*; XYY: *Populus simonii*; Z: algae crust; X: moss crust.

At the phylum level, the *nifH* diazotrophic communities in BSCs under the four sand-fixing plantations were assigned to 5 major phyla, and at the genus level to 10 major genera (Figure 4). The composition of the diazotrophic community (Figure 4a) shows that the Cyanobacteria and Proteobacteria are the dominant groups among BSCs. The combined relative abundance of the two ranges from 59.30% to 94.34%. Significant differences were observed in the composition of algae crust diazotrophic communities. In terms of relative abundance, the XYY plantations were dominated by Cyanobacteria (83.37%), in contrast to the NT plantations, where Proteobacteria was the predominant phylum (46.12%). The abundances of the two dominant phyla in the moss crust tended toward equilibrium, and differences between groups diminished. The Firmicutes was detected only in trace amounts in the moss crusts under the XYY plantations (1.10%), while the other low-abundance diazotrophic phyla were enriched in the moss crusts under the SL and WL plantations. The composition of the diazotrophic community showed (Figure 4b) that Skermanella was the dominant diazotrophic genus among BSCs under the SL, NT, and WL plantations. The relative abundance of this genus ranged from 10.94% to 44.36%. The Nostoc (Cyanobacteria) was significantly enriched in two types of crusts found under the XYY plantations (18.54% and 37.92%). The Mastigocladus is a key diazotrophic cyanobacterial group responsible for algae crust formation under NT and WL plantations (18.49% and 14.00%). A further comparison of diazotrophic community composition among plantation types is summarized below. The diazotrophic communities associated with algae crusts under SL, NT, and WL plantations are jointly dominated by Skermanella and Mastigocladus. Skermanella remains the dominant genus in the moss crust. In the moss crusts beneath the XYY plantations, the Nostoc completely replaced the previously dominant genus. The Azohydromonas, Geobacter, and Anaeromyxobacter are rare, low-abundance diazotrophic taxa found across all samples. Unannotated low-abundance diazotrophic communities (others) accounted for a higher proportion of samples from BSCs under SL, NT, and WL plantations. In summary, plantation types and BSC succession exert a hierarchical filtering effect on the diazotrophic community.

### 3.3. Test for Differences Between Groups at the Genus Level

**Figure 5:**
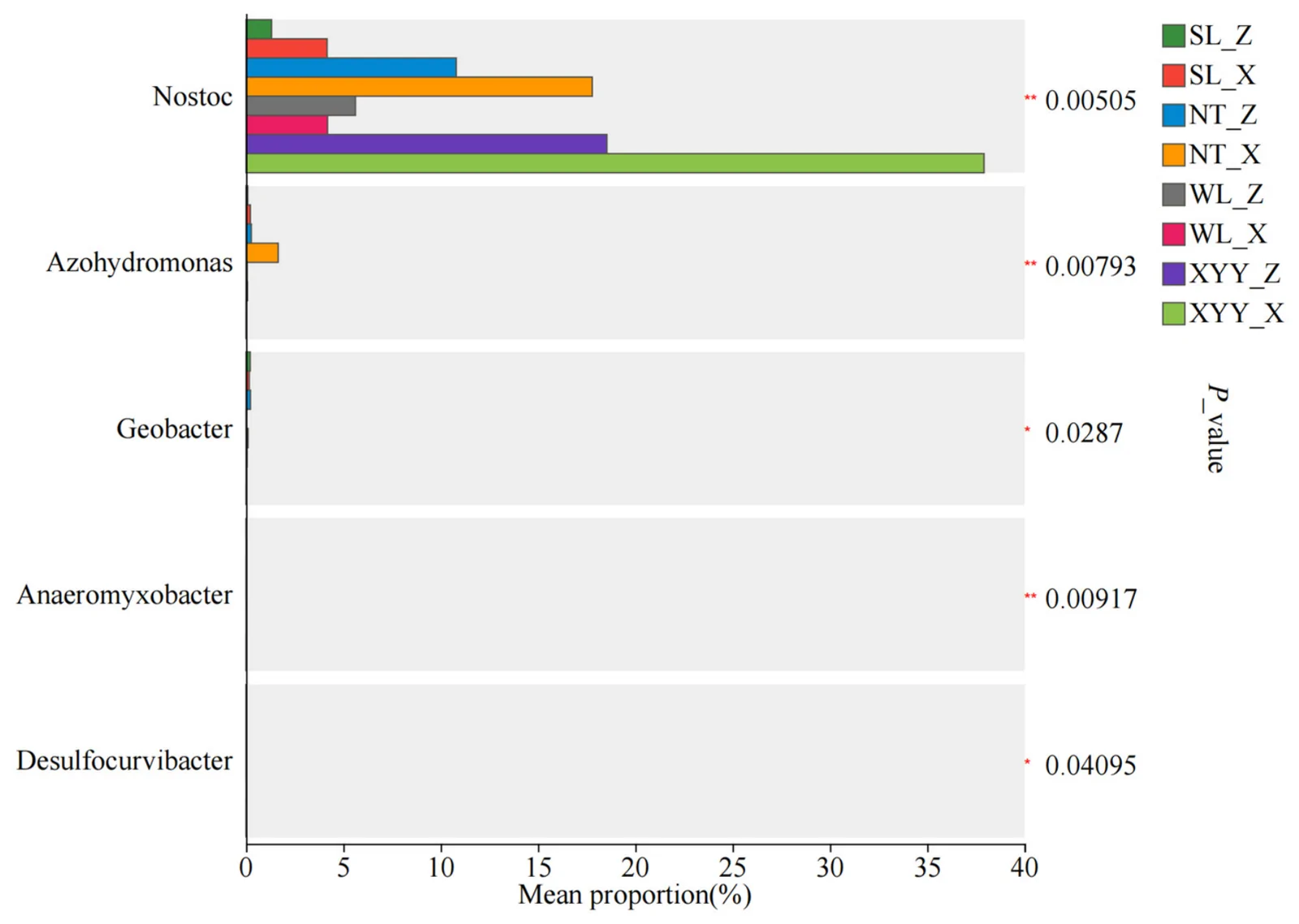
Test for differences among multispecies groups at the genus level. Note: the *p*-values for the tests of differences among the genus groups are shown on the right; SL: *Salix psammophila*; NT: *Caragana korshinskii*; WL: *Salix cheilophila*; XYY: *Populus simonii*; Z: algae crust; X: moss crust. Note: the P-values for the tests ofdifferences among the genusgroups are shown on the right; the asterisks in the figures indicatethe level of statistical significance (* *p* < 0.05, ** *p* < 0.01).

The relative abundances of diazotrophic genera in BSCs were analyzed across different plantations (Figure 5). The relative abundances of diazotrophic genera in BSCs varied significantly among the plantations. Among all samples, Nostoc was the dominant genus. Its highest average relative abundance (37.92%) occurred in the moss crust at the XYY plantations. The algae crust at the same site ranked second, with 18.54%. At the NT plantations, the average abundances in algae and moss crusts were 10.80% and 17.80%, respectively. In contrast, the abundances in both crust types at the SL and WL plantations were generally lower. The diazotrophic genus Azohydromonas had a very low overall abundance. A detectable proportion (1.65%) was found only in moss crusts of NT plantations. In algae and moss crusts of the other plantations, its average relative abundance was near zero. Geobacter, Anaeromyxobacter, and Desulfocurvibacter were organisms associated with anaerobic metabolism. Their abundances were low across all samples and nearly undetectable in most treatments. The *p*-values for intergroup differences were 0.0287, 0.0092, and 0.0410, respectively (rounded to three decimals). In summary, Nostoc was mainly enriched in moss crusts of the XYY plantations. In contrast, the other genera with significant differences were all present at low abundances in BSCs across the sand-fixing plantations in this region.

### 3.4. Alpha Diversity of the Diazotrophic Community in BSCs

**Figure 6:**
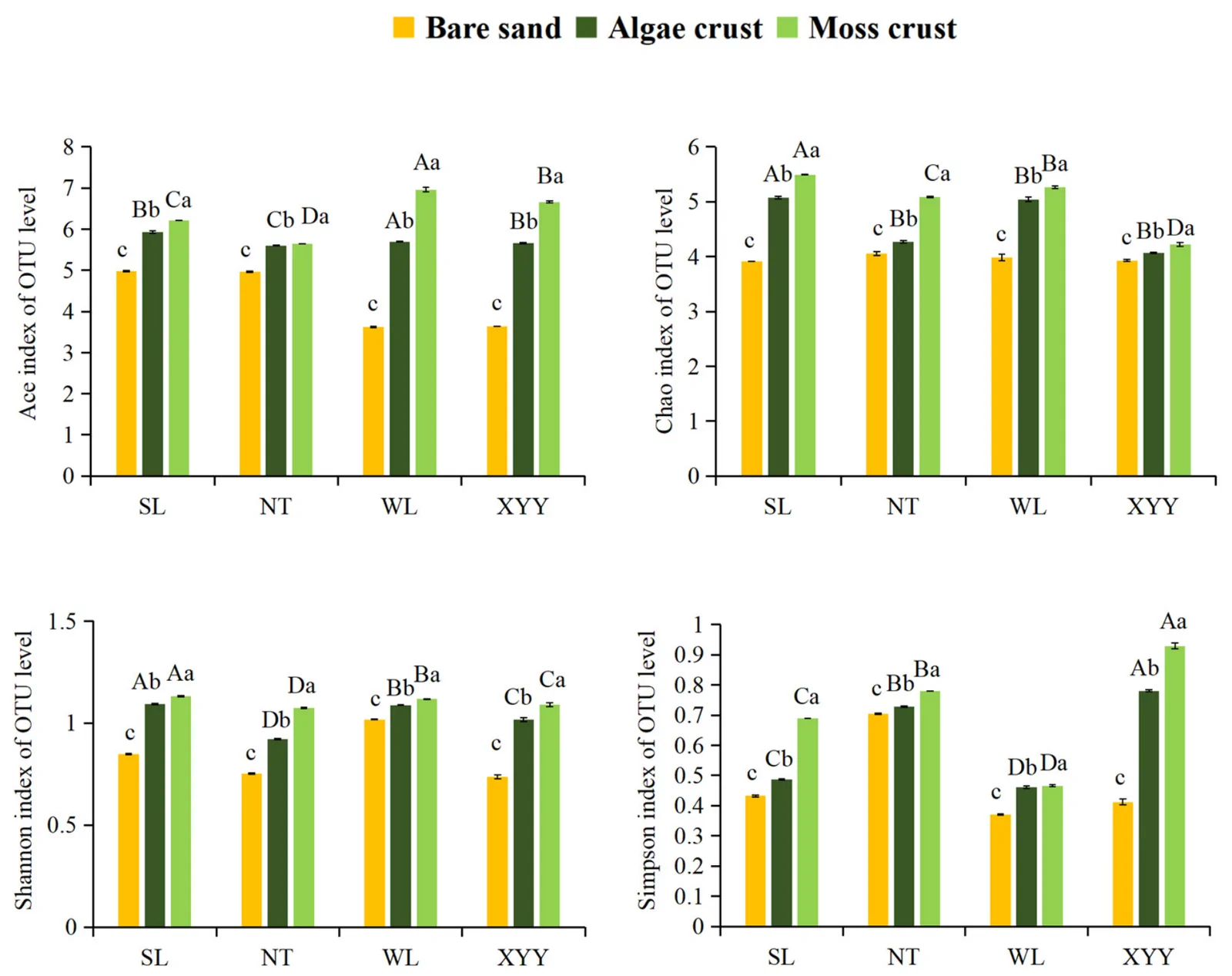
Diazotrophic community α-diversity in BSCs. Note: different uppercase letters indicate significant differences (*p* < 0.05) in the parameters of the same bark type across different plots, whereas different lowercase letters indicate significant differences (*p* < 0.05) in the parameters of different bark types within the same plot; SL: *Salix psammophila*; NT: *Caragana korshinskii*; WL: *Salix cheilophila*; XYY: *Populus simonii*.

Alpha diversity analysis revealed the richness and evenness of the *nifH* diazotrophic communities in BSCs under the four sand-fixing plantations (Figure 6). Both the abundance and diversity of algae crusts and moss crusts were significantly higher than those on bare sand (Ace: F_2,33_ = 52.22, *p* < 0.001; Chao1: F_2,33_ = 20.93, *p* < 0.001; Shannon: F_2,33_ = 31.01, *p* < 0.001; Simpson: F_2,33_ = 7.08, *p* = 0.003; partial η^2^ ranged from 0.30 to 0.76). As BSCs developed, both the species richness and evenness of the diazotrophic community increased significantly, as reflected by the Ace, Chao1, Shannon, and Simpson indices. The abundance order was as follows: moss crusts > algae crusts > bare sand. A further comparison of richness and evenness among plantation types is summarized below. At a given successional stage, plantation types significantly shaped the richness and evenness of diazotrophic communities. Across the BSC successional stages, the Ace index remained highest in the WL plantations (from 5.69 in algae crusts to 6.96 in moss crusts), indicating its superior species richness. The SL plantations consistently exhibited the highest Chao1 (increasing from 5.07 to 5.49) and Shannon (from 1.09 to 1.13) indices, suggesting the most even and diverse community structure. Meanwhile, the XYY plantations showed the highest Simpson index (rising from 0.78 to 0.93), reflecting a stronger predominance of dominant taxa. During the bare sand stage, differences in richness and evenness among plantation types were minimal. However, from the algae crust to the moss crust stage, the differentiation of diazotrophic communities among plantation types increased significantly. In summary, BSC succession significantly increased the richness and evenness of diazotrophic communities, with community differentiation also influenced by plantation types.

### 3.5. β-Diversity of Diazotrophic Community in BSCs

**Figure 7:**
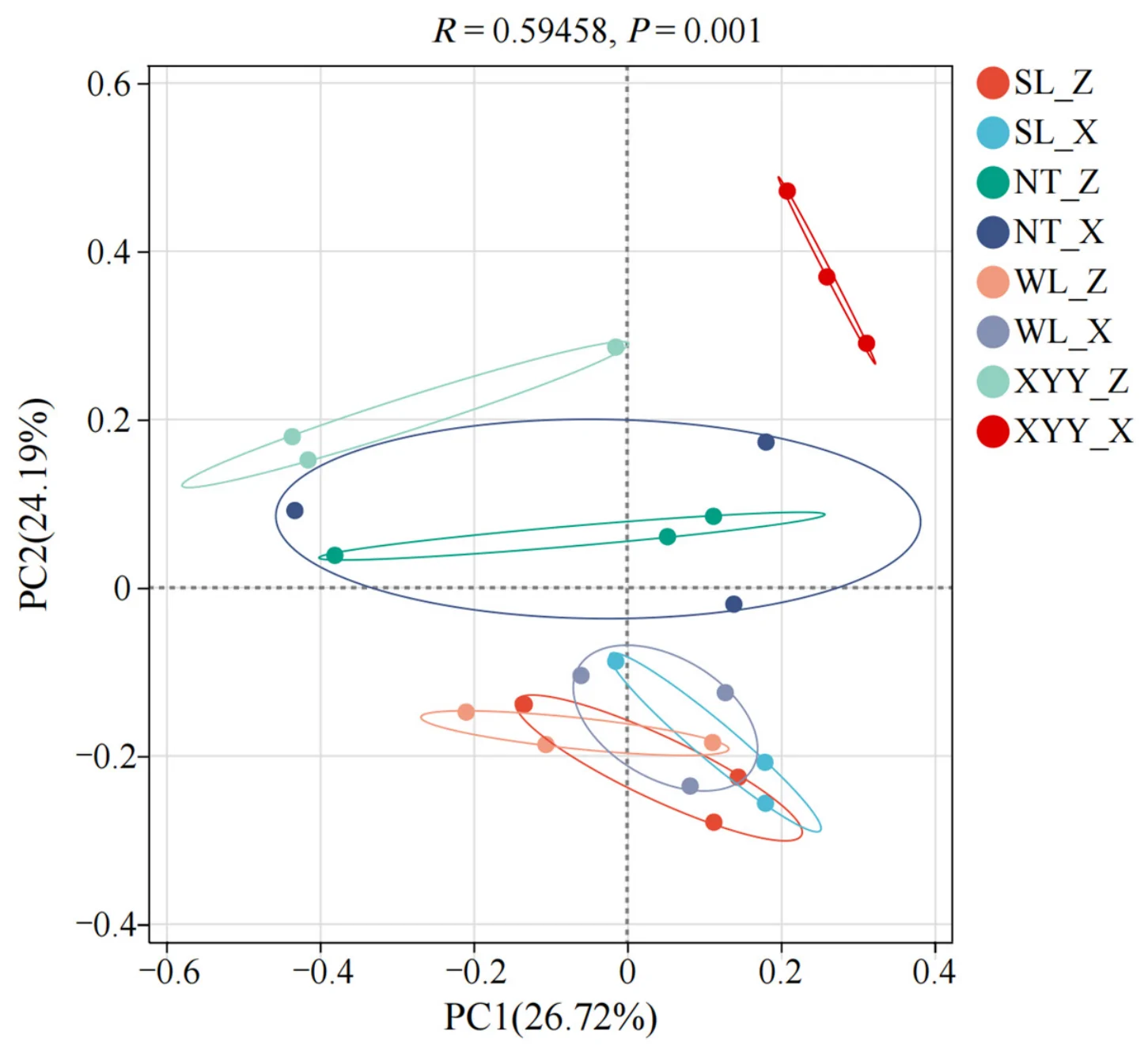
Principal coordinate analysis (PCoA) and ANOSIM test of diazotrophic community in BSCs. Note: similarity analysis ANOSIM results showed (*R* = 0.59458, *p* = 0.001); the ellipse represents the 95% confidence interval.; SL: *Salix psammophila*; NT: *Caragana korshinskii*; WL: *Salix cheilophila*; XYY: *Populus simonii*; Z: algae crust; X: moss crust.

PCoA clustering at the OTU level revealed that samples from algae and moss crusts under the four plantations formed distinct clusters (Figure 7). PC1 and PC2 explained 26.72% and 24.19% of the total variation, respectively, with a cumulative contribution of 50.91%. ANOSIM similarity analysis (*R* = 0.59458, *p* = 0.001) further confirmed that there were highly significant differences in the structure of diazotrophic communities among BSCs at different successional stages under various sand-fixing plantations. Samples from both crust types under the XYY plantations formed a distinct cluster, significantly separated from those under the SL, NT, and WL plantations. In contrast, a higher degree of overlap was observed between algae and moss crust communities under the SL and WL plantations, indicating greater similarity in species composition.

### 3.6. Correlation of Soil Environmental Factors and Diazotrophic Community in BSCs

#### 3.6.1. Correlation Analysis of Algae Crusts

**Figure 8:**
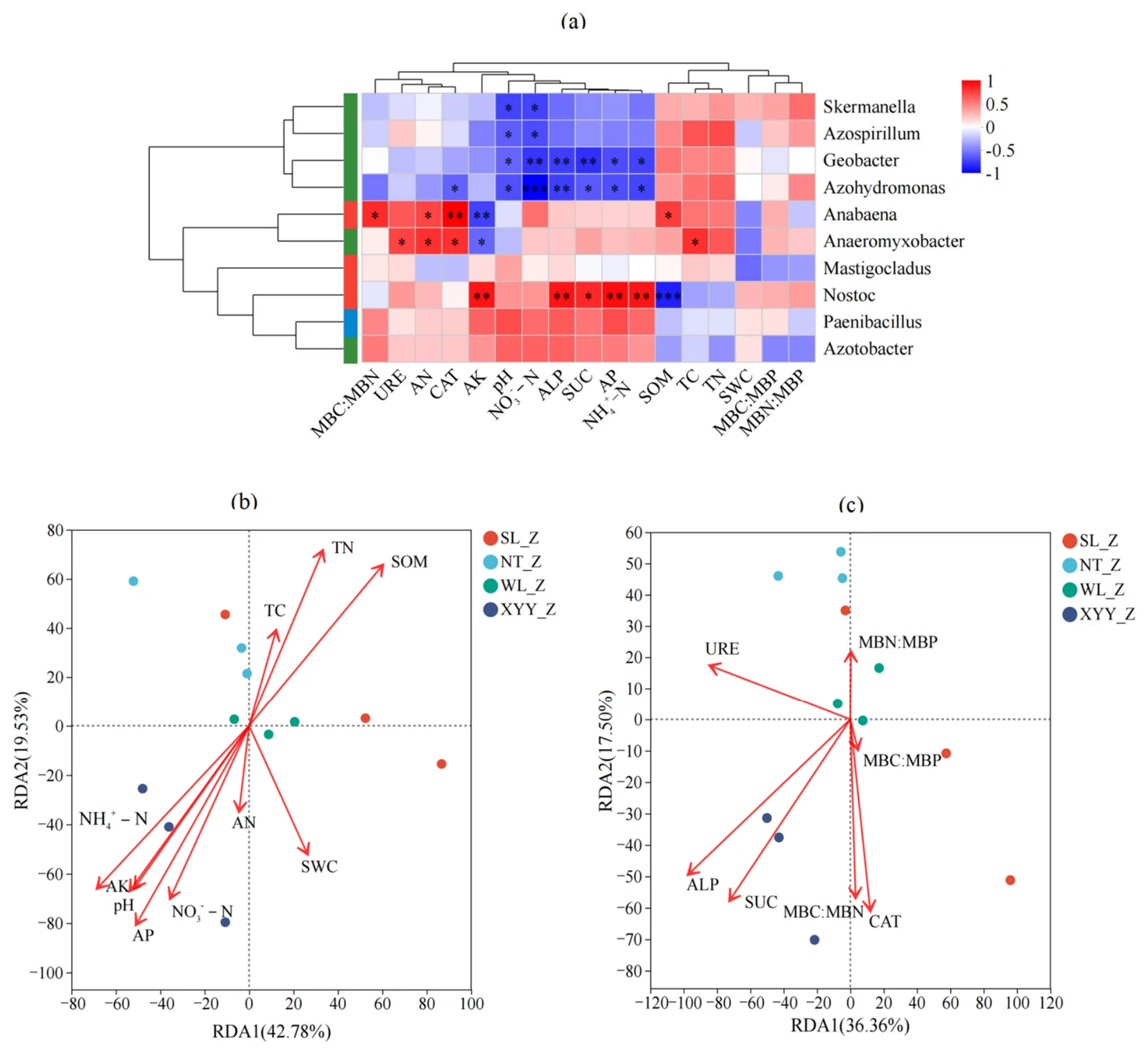
Analysis of environmental drivers of the diazotrophic community in algae crust. Note: (**a**) Spearman’s correlation heatmap of the diazotrophic community; (**b**) RDA of the diazotrophic community and soil physicochemical factors; (**c**) RDA of the diazotrophic community in relation to soil microbial biomass ratios and enzyme activity. The asterisks in the figures indicate the level of statistical significance (* *p* < 0.05, ** *p* < 0.01, *** *p* < 0.001); SL: *Salix psammophila*; NT: *Caragana korshinskii*; WL: *Salix cheilophila*; XYY: *Populus simonii*; Z: algae crust; SWC: soil water content; SOM: soil organic matter; AN: alkaline-hydrolyzable nitrogen; TC: total carbon; TN: total nitrogen; AP: available phosphorus; AK: available potassium; NH_4_^+^-N: ammonium nitrogen; NO_3_^−^-N: nitrate nitrogen; MBN: microbial biomass nitrogen; MBC: microbial biomass carbon; MBP: microbial biomass phosphorus; CAT: catalase; SUC: sucrase; URE: urease; ALP: alkaline phosphatase.

Spearman’s heatmap and redundancy analysis (RDA) consistently demonstrated that environmental factors significantly shaped the diazotrophic community structure in algae crusts (Figure 8). Spearman’s correlation analysis showed (Figure 8a) that the Anabaena was significantly positively correlated with MBC: MBN, AN, and SOM, and was extremely significantly positively correlated with CAT; the Anaeromyxobacter showed a significant positive correlation with URE, AN, CAT, and TC; the Nostoc showed a highly significant positive correlation with AK, ALP, AP, and NH_4_^+^-N, as well as a significant positive correlation with SUC; the Azohydromonas showed significant negative correlations with CAT, pH, NH_4_^+^-N, ALP, SUC, and AP; the Geobacter showed significant negative correlations with AK, pH, NO_3_^−^-N, ALP, SUC, and AP. Redundancy analysis (RDA) of the soil physicochemical factors showed (Figure 8b) that the RDA1 axis accounted for 42.78%, and the RDA2 axis accounted for 19.53%. Together, RDA1 and RDA2 explained 62.31% of the total variation in the diazotrophic community. RDA of the soil microbial biomass ratios, and enzyme activities (Figure 8c) showed that the RDA1 axis accounted for 36.36%, and the RDA2 axis accounted for 17.50%. Together, RDA1 and RDA2 explained 53.86% of the total variation in the diazotrophic community. SOM, AP, AK, NH_4_^+^-N, SUC, ALP, and URE were the key environmental factors associated with the differentiation of algae crust diazotrophic communities. The diazotrophic communities in algae crusts exhibited distinct environmental adaptation patterns across plantation types. In the NT plantations, communities are associated with high carbon and nitrogen content, and MBN:MBP stoichiometry. In the XYY plantations, communities are associated with high AK, AP, and ALP activity. In the SL plantations, communities are associated with CAT activity and MBC:MBP stoichiometry. In the WL plantations, communities are the least associated with environmental factors.

#### 3.6.2. Correlation Analysis of Moss Crusts

**Figure 9:**
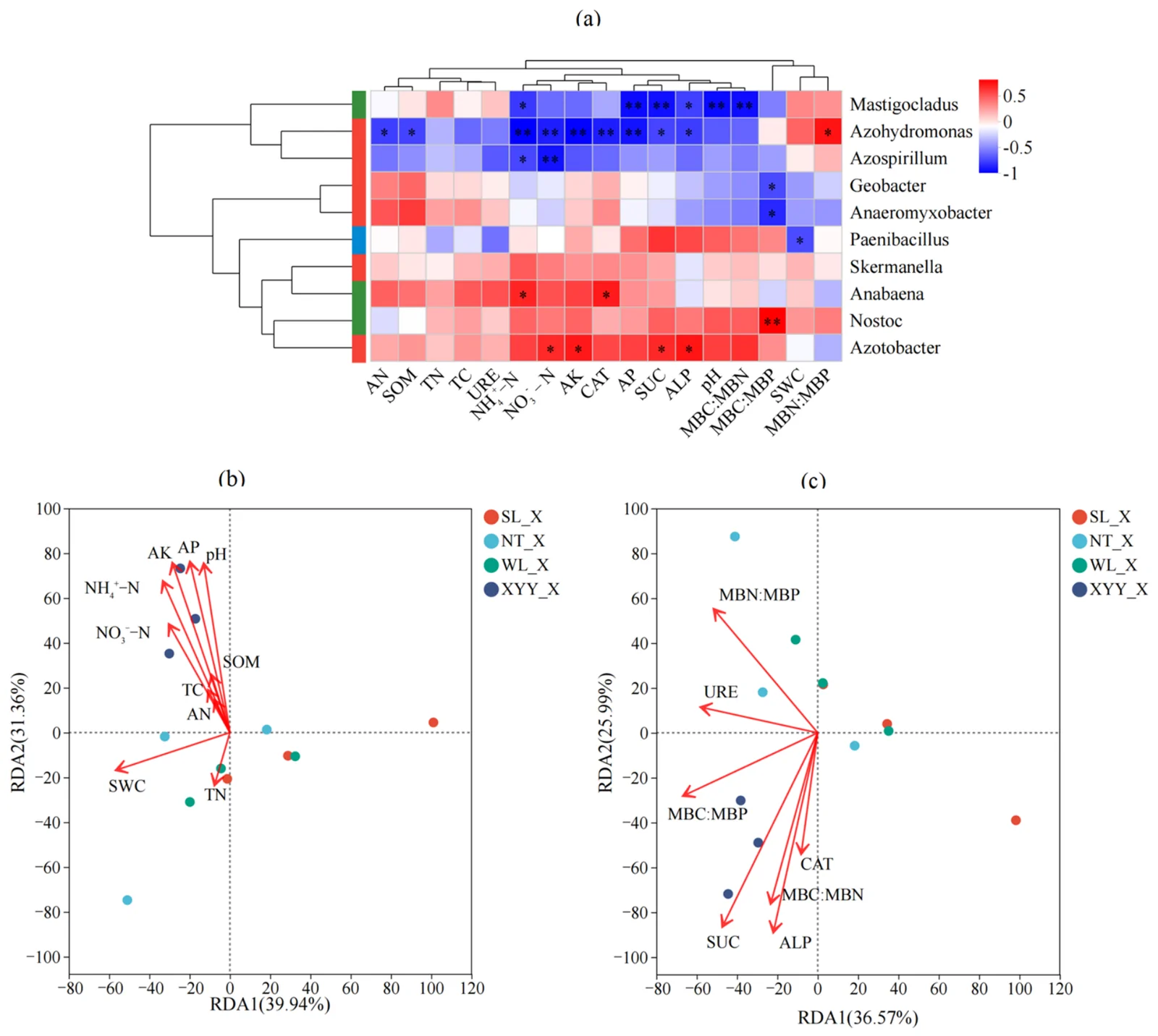
Analysis of environmental drivers of the diazotrophic community in moss crust. Note: (**a**) Spearman’s correlation heatmap of the diazotrophic community; (**b**) RDA of the diazotrophic community and soil physicochemical factors; (**c**) RDA of the diazotrophic community in relation to soil microbial biomass ratios and enzyme activity. The asterisks in the figures indicate the level of statistical significance (* *p* < 0.05, ** *p* < 0.01); SL: *Salix psammophila*; NT: *Caragana korshinskii*; WL: *Salix cheilophila*; XYY: *Populus simonii*; X: moss crust; SWC: soil water content; SOM: soil organic matter; AN: alkaline-hydrolyzable nitrogen; TC: total carbon; TN: total nitrogen; AP: available phosphorus; AK: available potassium; NH_4_^+^-N: ammonium nitrogen; NO_3_^−^-N: nitrate nitrogen; MBN: microbial biomass nitrogen; MBC: microbial biomass carbon; MBP: microbial biomass phosphorus; CAT: catalase; SUC: sucrase; URE: urease; ALP: alkaline phosphatase.

Spearman’s heatmap and RDA consistently demonstrated that environmental factors significantly shaped the diazotrophic community structure in moss crusts (Figure 9). The Spearman correlation heatmap (Figure 9a) compares diazotrophic correlations with biochemical indicators (e.g., physicochemical properties, microbial biomass). Moss crusts exhibited stronger correlations than algae crusts do across all sand-fixing plantations. The Azohydromonas showed a significant positive correlation only with the MBN:MBP, but highly significant negative correlations with NH_4_^+^-N, NO_3_^−^-N, AK, CAT, and AP; the Anabaena showed a significant positive correlation with NH_4_^+^-N and CAT; the Nostoc showed a highly significant positive correlation with MBC:MBP; and the Azotobacter showed a significant positive correlation with NO_3_^−^-N, AK, SUC, and ALP; the Mastigocladus showed a highly significant negative correlation with AP, SUC, pH, and MBC:MBN. RDA of the soil physicochemical factors showed (Figure 9b) that the RDA1 axis accounted for 39.94%, and the RDA2 axis accounted for 31.36%. Together, RDA1 and RDA2 explained 71.30% of the total variation in the diazotrophic community. RDA of the soil microbial biomass ratios and enzyme activities showed (Figure 9c) that the RDA1 axis accounted for 36.57%, and the RDA2 axis accounted for 25.99%. Together, RDA1 and RDA2 explained 62.56% of the total variation in the diazotrophic community. SWC, AP, AK, NH_4_^+^-N, MBN:MBP, and URE were identified as key environmental factors associated with the differentiation of moss crust diazotrophic communities. The diazotrophic communities in moss crusts exhibited distinct environmental adaptation patterns across plantation types. In NT plantations, communities are associated with high SWC, MBN: MBP, and URE activity. In XYY plantations, communities are associated with high pH, AP, TC, SOM, SUC, MBC: MBP, MBC:MBN, and high inorganic nitrogen. In SL plantations, communities are associated with high TN and CAT activity. In WL plantations, communities are the least associated with environmental conditions.

### 3.7. Functional Categories of the Diazotrophic Community in BSCs

**Figure 10:**
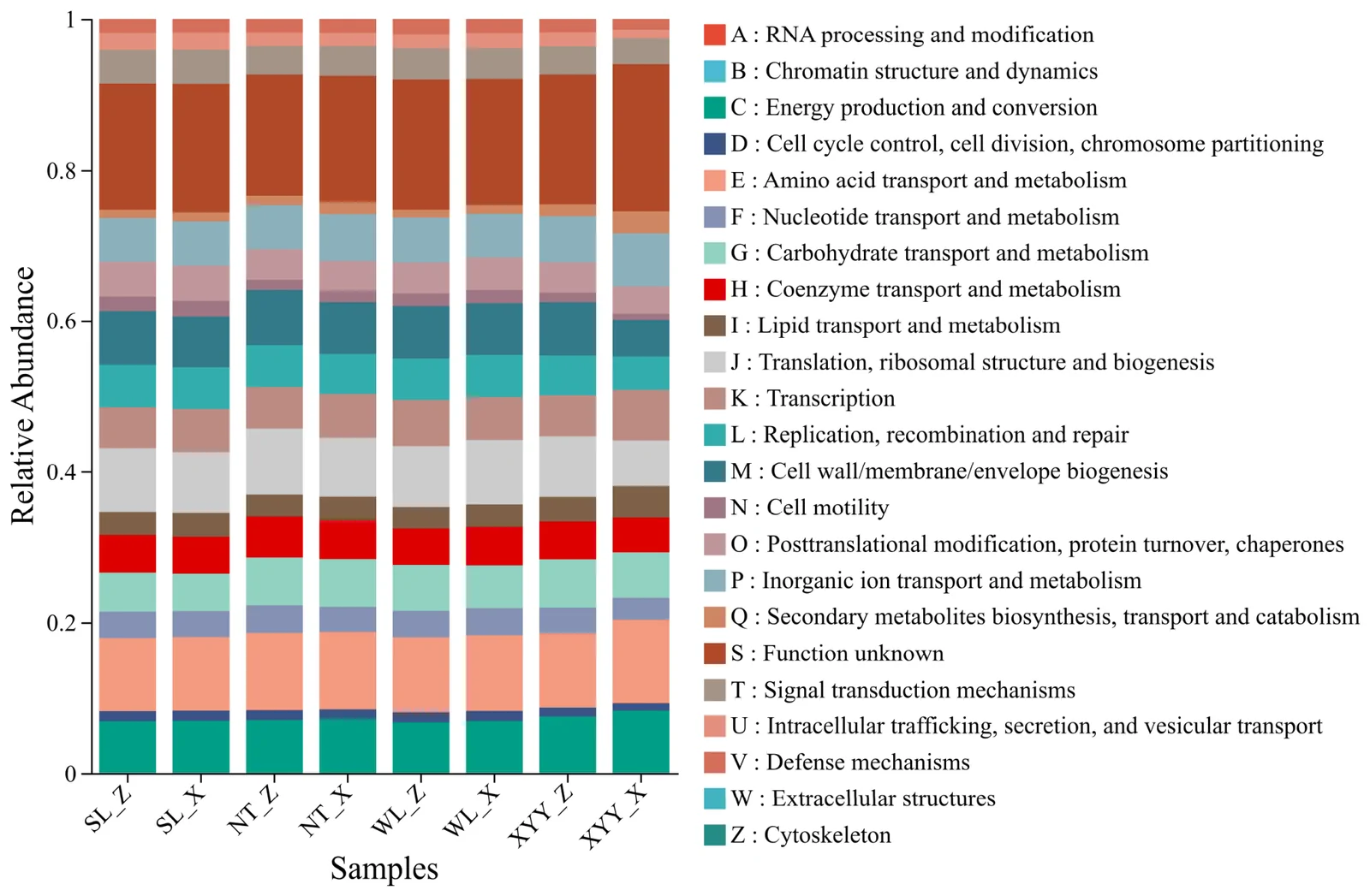
COG functional categories of diazotrophic communities in BSCs. Note: SL: *Salix psammophila*; NT: *Caragana korshinskii*; WL: *Salix cheilophila*; XYY: *Populus simonii*; Z: algae crust; X: moss crust.

Among all COG categories, proteins of unknown function (S) were the most abundant in every BSC sample from the sand-fixing plantations (Figure 10). Proteins of unknown function (S) was the most important functional components of diazotrophic communities (>16.00%). Translating ribosome synthesis (J), amino acid transport and metabolism (E), and energy production and conversion (C) were identified as the core fundamental functions of BSCs. The average relative abundances are 7.92%, 10.14%, and 7.11%, respectively. Together, they support the basic life processes and diazotrophic capacity of the diazotrophic communities in BSCs. Along the BSC successional gradient, the relative abundance of functional groups in moss crusts also converged with that in algae crusts. Functional differences among plantation types and successional stages were limited. In SL and WL plantations, the functional categories of COG in algae and moss crusts showed high convergence, and community functional stability was stronger; in NT plantations, amino acid metabolism (E) and energy metabolism pathways (C) were more active in algae crusts (8.62%), whereas carbohydrate transport metabolism (G) and coenzyme transport-related functions (H) were significantly enriched in moss crusts (5.68%). In XYY plantations, the proportion of two types of crust-associated proteins with unknown functions (S), as well as functions related to cell membranes (M) and inorganic ion transport (P), was higher than in the other plantation types.

## 4. Discussion

### 4.1. Differences in the Composition of Diazotrophic Community in BSCs

High-throughput *nifH* gene sequencing was used in this study. It characterized the diazotrophic community composition (phylum and genus levels) in algae and moss crusts from four sand-fixing plantations (SL, NT, WL, XYY). The results showed that vegetation type and BSC successional stage were jointly associated with significant differentiation in the diazotrophic community at both phylum and genus levels. This finding aligns with the reported patterns of diazotrophic community differentiation in BSCs from arid sandy ecosystems [43]. At the phylum level, Proteobacteria and Cyanobacteria dominated the diazotrophic communities in BSCs across the study area. This is consistent with results from multiple studies on BSCs in temperate sandy areas and deserts [2,4]. In algae crusts under SL, NT, and WL plantations, Proteobacteria was overwhelmingly dominant, whereas Cyanobacteria prevailed in those under XYY plantations. In moss crusts, the relative abundances of Proteobacteria and Cyanobacteria became more balanced. This indicates that the diazotrophic community in BSCs is shifting from a pattern dominated exclusively by cyanobacteria in the early stages of succession to one jointly dominated by cyanobacteria and Proteobacteria in the later stages of succession [44]. This change may be related to vegetation regulating soil nutrients and pH levels, thereby altering the relative abundance of the two microbial communities [11,45]. The Firmicutes were present at low abundances in all samples and were detected only in trace amounts in the moss crusts beneath the XYY plantations. This indicates that Firmicutes are not well-suited to proliferate in large numbers in the mature BSC habitats of plantations in the study area.

The diazotrophic community structure at the genus level further refined the community differentiation patterns within the dominant phyla. A notable feature of BSCs under SL, NT, and WL plantations is that Skermanella consistently dominated as the key diazotrophic genus across all three plantation types. This genus also represents a core diazotrophic taxon commonly found in BSCs across temperate desert ecosystems [46]. Among them, Mastigocladus was enriched in the algae crusts under NT plantations. This indicates that the micro-environmental conditions beneath NT plantations are conducive to the colonization of Mastigocladus [4]. In the BSCs of SL and WL plantations, low-abundance anaerobic diazotrophic genera such as Geobacter, Anaeromyxobacter, and Azohydromonas accounted for a higher proportion. This indicates that a sandy, relatively nutrient-poor microenvironment is more conducive to the colonization of a rare anaerobic diazotrophic community [11]. The diazotrophic communities of BSCs growing under the XYY plantations exhibit distinct characteristics. The Nostoc is the signature dominant group and is absolutely dominant in the moss crusts. This indicates that Nostoc prefers nutrients with high availability and a slightly alkaline microenvironment [11]. The BSC development is associated with shifts in diazotrophic community composition. Within the same plantation type, algae crust communities exhibited stronger convergence, whereas community divergence became significantly amplified upon succession to the moss crust stage. This indicates that the development of BSCs amplifies community differentiation within diazotrophic communities under different vegetation types. This is consistent with the findings from studies of the wind-and-sand areas of the Loess Plateau [11]. Based on the diazotrophic community composition at both the phylum and genus levels, vegetation type emerged as a key factor shaping the diazotrophic community in BSCs. The development of BSCs further exacerbates community differentiation among diazotrophic communities across different forest sites at the phylum and genus levels. This difference is related to tree species biological traits (litter quality, diazotrophic capacity, and canopy structure), which alter the soil carbon and nitrogen baseline, understory light availability, and BSC microenvironment, thereby influencing the differential selection of diazotrophic communities [47,48].

### 4.2. Responses of Diazotrophic Community Diversity and Structure in BSCs

Based on α-diversity indices derived from high-throughput sequencing of the *nifH* gene and PCoA-ANOSIM analysis, this study indicates the differential patterns of species diversity and overall community structure of diazotrophic communities in algae and moss BSCs in relation to vegetation types. This finding is consistent with the results of most mainstream studies [10,14]. The Chao1, Ace, and Shannon indices for algae and moss crusts under the SL and WL plantations were higher than those under the XYY plantations, while the Simpson index was lower. This indicates that diazotrophic communities under these two shrub plantations exhibit higher species richness and evenness [49]. The Chao1 and Shannon indices for algae and moss crusts under the XYY plantations were the lowest across all treatments, while the Simpson index was the highest. This indicates the diazotrophic community characterized by reduced species richness, limited overall diversity, and strong dominance by a few taxa (such as Nostoc). This is related to the fact that high levels of available phosphorus and potassium, combined with inorganic nitrogen, lead to the directional enrichment of a single dominant diazotrophic group in the environment, thereby significantly reducing community species evenness [46,50,51]. During BSC succession, moss crusts generally exhibited higher α-diversity than algae crusts within the same plantation type. This is related to the accumulation of organic matter on the surface of the moss crust and the heterogeneity of the microhabitat, which enhance the supply of carbon and nitrogen substrates, thereby increasing the diversity of the diazotrophic community [4,52].

The PCoA ordination results show that samples of algae and moss crusts collected from the same plantation cluster together in the ordination space. In contrast, samples from different plantations form distinct clusters. This indicates that vegetation type is an important environmental selection factor associated with the overall differentiation of diazotrophic communities [53,54]. Axes PC1 and PC2 together account for 50.91% of the total variation in the diazotrophic community. This indicates that vegetation type has a strong, though not exclusive, shaping effect on the structure of the biological soil crust diazotrophic community. ANOSIM analysis (*R* = 0.59458, *p* = 0.001) further confirmed highly significant differences in diazotrophic community structure among the four plantation types. Among these, samples of BSCs collected under the XYY plantations formed a distinct cluster, and their community characteristics differed significantly from those of other plantations. This indicates that the diazotrophic communities in the XYY plantation BSCs exhibit the highest degree of differentiation. This also corresponds exactly to the community composition at the phylum and genus levels. The diazotrophic communities associated with the two types of crusts under NT plantations showed a higher degree of overlap and greater similarity in species composition. This is related to the soil carbon and nitrogen baseline improved by leguminous shrubs, which shapes the unique diazotrophic community structure of these shrubs [55]. As BSCs developed, algae crust samples from the same plantation type exhibited tighter clustering, whereas moss crust samples displayed greater dispersion. This indicates that the development of BSCs enhances the environmental influence of vegetation on diazotrophic communities [11]. A synthesis of the results from α-diversity and PCoA-ANOSIM analyses revealed that the type of sand-fixing plantation and the succession of BSCs exerted a significant influence on the diazotrophic community. This influence was consistent with the compositional differences observed at both the phylum and genus levels among plantation types and successional stages.

### 4.3. Environmental Correlates of Diazotrophic Community Structure in BSCs

Based on a Spearman’s correlation heatmap of the *nifH* diazotrophic genera and soil physicochemical and microbial metabolic indicators. It clarifies the species-specific relationships between each dominant diazotrophic genus in BSCs and individual environmental factors. Combined with two sets of RDA results, the comprehensive factors influencing diazotrophic community differentiation were analyzed at the community-wide scale. The results are highly consistent with the findings of numerous studies on BSCs in the desert [4,56,57]. Spearman’s correlation analysis revealed that, during the algae crusts stage, environments with high carbon-to-nitrogen ratios and high hydrolytic enzyme activity positively enriched Nostoc and Anabaena, while strongly inhibiting Azohydromonas. High pH, high available phosphorus and potassium, and high nitrogen and phosphorus-hydrolyzing enzyme activity significantly inhibited the distribution of Geobacter. These results indicate that, during the algae crust stage, the differential distribution of diazotrophic taxa is closely associated with soil stoichiometry and enzymatic dynamics [13]. During the moss crust stage, carbon and nitrogen nutrients, in synergy with hydrolases, promote the enrichment of dominant diazotrophic genera such as Nostoc and Azotobacter, while simultaneously inhibiting Mastigocladus and Azohydromonas. This indicates the presence of a clear nutrient-selective effect during the late stages of succession [11,13]. This difference was associated with multiple factors. Carbon and nitrogen substrates, nutrient availability, pH, and extracellular hydrolases all exerted selective effects on diazotrophic taxa. In addition, the microenvironmental differences between algae crusts and moss crusts contributed to distinct niche differentiation patterns of diazotrophic communities [4,11,13].

RDA of soil physicochemical properties revealed that the first two axes cumulatively explained over 60% of the community variation in both algae and moss crust stages, with higher explanatory power observed in the moss crust stage. This result is consistent with the findings of most BSC studies [4,33,35]. This indicates that as BSCs transition from the algae to the moss stage, the coupling between diazotrophic communities and environmental factors increases [57,58,59]. The diazotrophic communities in algae and moss crusts were associated with different combinations of environmental factors, and these associations also differed between the two crust types within the same plantation type. Algae crust formation is primarily regulated by SOM, AP, AK, NH_4_^+^-N, while moss crust formation is influenced by a combination of SWC, AP, AK, and NH_4_^+^-N. This indicates that BSC succession is accompanied by systematic changes in key environmental factors related to diazotrophic community assembly. These changes include continuous modifications in soil nutrients and moisture [11,60]. RDA of soil microbial biomass ratios and enzyme activities revealed that the first two axes explained 53.86% of the diazotrophic variation during the algae crust stage. The moss crust stage accounted for 62.56% of the total variation in the community. This further indicates that, as BSCs develop, the coupling between the diazotrophic community and environmental factors becomes stronger [57,58,59]. SUC, ALP, and URE are the key factors influencing the diazotrophic community in algae crusts. MBN:MBP and URE are the key factors influencing the diazotrophic community in moss crusts. This indicates a fundamental shift in the patterns of diazotrophic community assembly during BSC succession, corresponding to a transition from resource dependence to homeostasis [44,61]. Further analysis of the differential patterns observed in plantations revealed that algae and moss crusts are regulated by different soil environmental factors in terms of their diazotrophic communities. This indicates that the composition of the diazotrophic community is associated with BSC succession and environmental factors related to plantation types [62]. Comparing the dispersion of RDA ordination between the two crust types, moss crust samples exhibited greater dispersion. This indicates that during the moss crust stage, environmental factors are more closely related to diazotrophic communities, and diazotrophic community differences among plantation types are more evident [63,64]. This is related to the fact that sand-fixing plantations influence the structure of diazotrophic bacterial communities by first altering the physicochemical properties of BSCs and then affecting microbial metabolic activity [54,65].

### 4.4. Differences in Functional Categories of Diazotrophic Community in BSCs

COG functional categories showed a consistent pattern across all sites. Translating ribosome synthesis (J), amino acid transport and metabolism (E), and energy production and conversion (C) were the dominant groups. They consistently accounted for the largest proportions. This indicates that various soil diazotrophic communities have biological nitrogen fixation as their core metabolic characteristic [66]. The proportion of components related to photosynthetic nitrogen fixation and inorganic nitrogen transport was significantly higher in algae and moss crusts under XYY plantations. This indicates that tree species possess a highly efficient capacity for inorganic nitrogen transport and utilization [61]. This finding also confirms, at the functional gene level, that plantation type is an important factor associated with the functional differentiation of diazotrophic communities in BSCs. The proportion of components related to heterotrophic nitrogen fixation in BSCs growing under SL, WL, and NT shrub plantations was particularly high. This indicates that the diazotrophic function of BSCs under shrub stands is associated with not only photosynthetic nitrogen fixation by cyanobacteria but also heterotrophic nitrogen fixation. This is related to the fact that the shrub canopy provides a suitable microenvironment and carbon source for heterotrophic diazotrophic bacteria through shading and litterfall [49,67]. This difference also reflects the divergent effects of shrub versus tree plantations on the nitrogen fixation pathways of BSCs. As BSCs developed, the relative abundance of overall functional groups in moss crusts converged with that in algae crusts. The proportions of COG categories related to substance transport, energy metabolism, and stress response also converged. This indicates that BSCs undergo functional convergent evolution under long-term environmental conditions [68]. Based on the COG functional category results, plantation type, soil physicochemical properties, and microbial metabolism were jointly associated with the community structure and functional gene composition of diazotrophic communities. As BSCs developed, the functional components involved in nitrogen fixation and the nitrogen cycle tended to converge across different plantation types and successional stages. This is related to the systematic changes in soil physicochemical properties (pH, organic matter, total nitrogen, etc.) associated with plantation type and BSC succession, the dominance of Nostoc in both crust types, and the consequent convergence of functional components [2,4,11].

Although *nifH* amplicon sequencing cannot capture gene expression, functional activity, or genomic context, it remains a reliable marker for diazotrophic community profiling. Future studies should combine direct activity assays, such as the acetylene reduction assay (ARA) or ^15^N isotope tracer experiments, with transcriptomic analyses to distinguish gene presence from actual expression. Such integrated approaches would help clarify the factors contributing to differences in biological nitrogen fixation (BNF), and guide the selection of sand-fixing plantations for alpine sandy land restoration.

## 5. Conclusions

In this study, we comprehensively analyzed the community structure, taxonomic composition, functional gene characteristics, and environmental associations of diazotrophic communities under different sand-fixing plantation types and across BSC successional stages. The main conclusions are as follows: (1) At the phylum level, Proteobacteria and Cyanobacteria were the two dominant phyla in diazotrophic communities of BSCs growing under sand-fixing plantations on alpine sandy land; at the genus level, diazotrophic community composition varied across plantation types. In the SL, NT, and WL plantations, Skermanella was the dominant genus. In the XYY plantations, Nostoc was the core dominant diazotrophic genus. In the NT and WL plantations, Mastigocladus was identified as a key taxon. (2) The α-diversity results show that the diversity of diazotrophic communities in BSCs under tree plantations (XYY) is significantly lower than that in three types of shrub plantations (SL, NT, WL). PCoA-ANOSIM analysis confirmed a clear boundary between tree and shrub plantation communities, with moss crust diazotrophic communities showing greater differentiation than algae crust communities. (3) Spearman’s correlation analysis and RDA ordination results suggest that diazotrophic community differentiation is associated with different sand-fixing plantations, as well as with soil physicochemical properties and microbial metabolic characteristics. The degree of differentiation in moss crust communities resulting from environmental selection is significantly higher than that in algae crusts. The diazotrophic communities in algae and moss crusts were associated with different sets of soil environmental factors. In algae crusts, SOM, AP, AK, NH_4_^+^-N, SUC, ALP, and URE were identified as major correlates; in moss crusts, SWC, AP, AK, NH_4_^+^-N, MBN: MBP, and URE were the primary correlates. (4) COG functional annotation revealed that proteins with unknown function (S) constituted the most abundant functional component of diazotrophic communities. Translating ribosome synthesis (J), amino acid transport and metabolism (E), and energy production and conversion (C) represented the core foundational functions of BSCs across all sites. As BSCs developed, the functional components involved in nitrogen fixation and the nitrogen cycle tended to converge across different plantation types and successional stages.

In summary, this study investigated the composition and diversity of diazotrophic communities within BSCs across different plantations in alpine sandy land, along with their correlations with soil environmental factors and functional profiles based on COG categories. A framework of associations has been observed, linking vegetation and soil environments to the structure and composition of diazotrophic communities and their nitrogen metabolism functions. These findings further reflect the adaptive strategies of diazotrophic communities to the alpine sandy land, as well as the regulatory roles of environmental factors in shaping community structure. Therefore, in desertification control and ecological restoration practices, sand-fixing tree species should be selected and combined according to local environmental conditions. Furthermore, restoration strategies should be optimized based on key soil environmental factors, with the aim of enhancing the stability of artificial ecosystems and securing long-term restoration outcomes through the targeted regulation of diazotrophic communities.

## Figures and Tables

**Table 1:** Basic information on the sample plot.

Sample Plot	Abbreviation	Latitude and Longitude	Elevation (m)	Vegetation Cover (%)	Dominant Vegetation
*Salix psammophila*	SL	36°14′47.35″ N, 100°14′23.39″ E	2879.1	76	*Leymus secalinus, Astragalus adsurgens* et al.
*Caragana korshinskii*	NT	36°14′46.85″ N, 100°14′21.78″ E	2876.2	85	*Leymus secalinus, Artemisia sphaerocephala* et al.
*Salix cheilophila*	WL	36°14′46.27″ N, 100°14′23.60″ E	2877.6	80	*Hippophae rhamnoides, Thermopsis lanceolata, Caragana intermedia* et al.
*Populus simonii*	XYY	36°14′45.69″ N, 100°14′27.41″ E	2874.9	68	*Hippophae rhamnoides*, *Artemisia sphaerocephala* et al.

Note: SL, NT, WL, and XYY denote *Salix psammophila*, *Caragana korshinskii*, *Salix cheilophila*, and *Populus simonii* plantations, respectively. Coordinates are in WGS 84 (DMS). Vegetation cover was estimated by the quadrat method (60 m × 60 m). All sites are within the Qinghai Desertification Control Experimental Station.

**Table 2:** Basic information on samples.

Crust Type	Index	*Salix psammophila* (SL)	*Caragana korshinskii* (NT)	*Salix cheilophila* (WL)	*Populus simonii* (XYY)
Bare sand	Crust thickness (mm)	-	-	-	-
	Crust coverage (%)	-	-	-	-
Algae crust	Crust thickness (mm)	5.89 ± 1.13 Cb	5.75 ± 0.85 Db	7.74 ± 0.36 Ab	7.15 ± 1.09 Bb
	Crust coverage (%)	16.12 ± 6.10 Cb	18.46 ± 3.02 Bb	31.72 ± 4.13 Ab	30.62 ± 12.38 Ab
Moss crust	Crust thickness (mm)	7.42 ± 1.36 Da	10.16 ± 1.61 Ca	12.21 ± 1.43 Aa	12.14 ± 3.12 Ba
	Crust coverage (%)	29.61 ± 7.45 Da	35.05 ± 4.33 Ca	40.25 ± 10.74 Aa	39.39 ± 7.19 Ba

Note: “-” indicates that it has no meaning. Different uppercase letters indicate significant differences (*p* < 0.05) in the parameters of the same bark type across different plots, whereas different lowercase letters indicate significant differences (*p* < 0.05) in the parameters of different bark types within the same plot.

## Data Availability

The raw *nifH* gene amplicon sequencing data generated in this study have been deposited in the NCBI Sequence Read Archive (SRA) under BioProject accession number PRJNA1509164. The original contributions presented in this study are included in the article. Further inquiries can be directed to the corresponding authors.

## Abbreviations

The following abbreviations are used in this manuscript:

BSCs	Biological soil crusts
PCoA	Principal coordinate analysis
ANOSIM	Analysis of Similarities
RDA	Redundancy Analysis
PCR	Polymerase Chain Reaction
SL	*Salix psammophila*
NT	*Caragana korshinskii*
WL	*Salix cheilophila*
XYY	*Populus simonii*
SWC	soil water content
SOM	soil organic matter
TN	total nitrogen
TC	total carbon
AP	available phosphorus
AK	available potassium
MBC	microbial biomass carbon
MBN	microbial nitrogen
MBP	microbial phosphorus
CAT	catalase
URE	urease
SUC	sucrase
ALP	alkaline phosphatase
COG	Clusters of Orthologous Groups of proteins
